# Exploring the relationship between performance anxiety and subjective well-being in music performance students: the role of psychological resilience and perfectionism

**DOI:** 10.3389/fpsyg.2026.1967733

**Published:** 2026-10-26

**Authors:** Xiong Gao, Pingting Li

**Affiliations:** 1 Department of Student Affairs, Zhangjiajie College, Zhangjiajie, Hunan, China; 2 School of Art, Zhangjiajie College, Zhangjiajie, Hunan, China

**Keywords:** adaptive perfectionism, maladaptive perfectionism, performance anxiety, psychological resilience, subjective well-being

## Abstract

**Background:**

Music performance students frequently encounter high-evaluation training and performance contexts, yet the conditional associations among performance anxiety, psychological resilience, perfectionism, and subjective well-being remain insufficiently understood.

**Methods:**

A cross-sectional survey was conducted among 946 university music performance students in China. PROCESS Model 4 examined the indirect association through psychological resilience, while Model 72 examined the separate and joint moderating roles of adaptive and maladaptive perfectionism.

**Results:**

Performance anxiety was negatively associated with subjective well-being. A significant indirect association was observed through psychological resilience. Adaptive and maladaptive perfectionism each moderated the performance anxiety-resilience association, whereas their three-way interaction was not significant. Both perfectionism dimensions also moderated the positive resilience-well-being association. The three-way interaction on this association was significant and negative, while the resilience-well-being association remained positive across all examined combinations of perfectionism.

**Conclusion:**

Psychological resilience statistically accounted for part of the association between performance anxiety and subjective well-being, while adaptive and maladaptive perfectionism showed distinct separate and joint conditional patterns across the two focal associations.

## Introduction

Over the past few years, the frequency of evaluation and the intensity of stage performance in higher education art programs have continued to increase. Music performance students frequently shift between coursework, rehearsals, and public performances, immersing themselves in a training environment characterized by both high arousal and high evaluation. Subjective well-being (SWB), which encompasses individuals’ affective experiences and cognitive evaluations of life satisfaction (Diener, 1984), is an important indicator of how individuals experience and evaluate their lives and is relevant to understanding health and adjustment (Das et al., 2020). Attention to SWB complements the assessment of psychological distress, as positive mental health cannot be inferred solely from the absence of symptoms (Keyes, 2002). Music performance students frequently report lower levels of health and happiness than students in other majors (Alessandri et al., 2020). Within performance contexts, music performance anxiety (PA) refers to apprehension associated with anticipated or actual musical performance, involving cognitive, physiological, and behavioral responses, such as worry about mistakes, bodily tension, and avoidance (Bakhtiari et al., 2025). It is a common experience closely tied to judgment and self-expectations. Research has consistently shown that music PA is associated with greater psychological distress and lower SWB (Kinney et al., 2025; Miksza et al., 2021; Wang and Yang, 2024). Therefore, focusing on the SWB of music performance students from the perspective of a high-evaluation training environment carries both practical and educational significance.

Research on the factors associated with SWB among music performance students has provided complementary evidence from three perspectives: stress, psychological resources, and individual differences. First, from the stress perspective, music PA shows stable associations with negative self-appraisal, post-performance rumination, and threat-based evaluations, and it correlates with lower SWB and poorer quality of life. These findings highlight the close connection between stress experiences in evaluative contexts and levels of well-being (Guyon et al., 2020; Jiang and Tong, 2024). Second, from the psychological perspective, according to resilience theory, psychological resilience (PR) is conceptualized as a process-oriented capacity associated with maintaining or regaining adaptive functioning under stress or adversity. Greater psychological resilience has been associated with more favorable emotional adjustment, including fewer symptoms of psychological distress among musicians (Kegelaers et al., 2021). In music performance student samples, PR has shown a positive association with SWB, and previous studies have also reported close associations between resilience and other psychological resources, such as self-efficacy (de Bruin, 2025; Feng and Wang, 2025; Tu and Fu, 2024). Third, from the perspective of individual differences, perfectionism is particularly relevant to music performance, where exacting performance demands and frequent evaluation place personal standards and responses to perceived shortcomings at the center of students’ experiences. Adaptive perfectionism (AP) emphasizes high personal standards and achievement striving, whereas maladaptive perfectionism (MP) involves self-critical evaluations and dissatisfaction with performance (Stoeber and Otto, 2006). Among musicians, perfectionistic striving has been associated with greater effort and achievement, while negative reactions to imperfection have been associated with greater distress (Stoeber and Eismann, 2007). The relationship between high standards and music PA also varies across studies (He and Ding, 2025; McNeil et al., 2022). Taken together, music PA, PR, and multidimensional perfectionism constitute key factors for understanding differences in SWB among music performance students.

Recent intervention research has considered both music performance anxiety and positive psychological functioning, highlighting the relevance of examining distress alongside positive outcomes in musical contexts (Zhang et al., 2026). Although relevant findings are accumulating, few studies have systematically integrated PA, PR, and perfectionism within the same model to explain SWB in music performance students. In order to close this gap, the current study adopts resilience theory (Masten, 2001; Sarkar and Fletcher, 2014) and multidimensional perfectionism theory (Stoeber and Otto, 2006) as an integrative framework. Using a sample of university-level music performance students, we investigate the relationship between anxiety related to performing music and SWB, examine the indirect role of PR, and examine whether AP and MP condition the focal associations. This study addresses an important research gap and provides correlational evidence that may inform future research and psychological support practices for music performance students.

## Theoretical basis

This study adopts resilience theory (Masten, 2001; Sarkar and Fletcher, 2014) as its core framework. Resilience is conceptualized as a process-oriented capacity associated with maintaining or regaining adaptive functioning under stress or adversity (Morgan et al., 2017; Sarkar and Fletcher, 2014). Resilience concepts have gained popularity recently in domains like education, mental health, and athletic performance (Abulfaraj et al., 2024; Schäfer et al., 2024; Wang et al., 2025). In keeping with this perspective, recent reviews of music PA have highlighted links between stage-related stressors, coping strategies, and mental health outcomes, thereby providing external support for applying a resilience framework to the context of music performance (Twitchell et al., 2025). Accordingly, PR is regarded as an important psychological resource that may statistically explain a portion of the correlation between PA and SWB among music performance students.

Multidimensional perfectionism theory complements this framework by linking personal standards and self-evaluation to psychological adjustment (Frost et al., 1990; Hewitt and Flett, 1991; Stoeber and Otto, 2006). AP emphasizes high personal standards, while MP captures dissatisfaction with perceived performance discrepancies. These tendencies may condition how PA relates to perceived coping capacity and how PR relates to emotional experience and life satisfaction. The 2 × 2 model further emphasizes differences in adjustment across combinations of perfectionism dimensions (Gaudreau and Thompson, 2010). Together, these frameworks provide a basis for examining the separate and joint moderating roles of AP and MP on the PA-PR and PR-SWB associations, with potentially different patterns across the two associations.

## Review and hypotheses

### Performance anxiety and subjective well-being

PA is a common psychological difficulty among music performance students, often manifested as tension, worry, and self-doubt in performance or evaluative contexts (Barros et al., 2022). Existing research repeatedly demonstrates that lower life satisfaction and wellbeing are linked to increased anxiety levels (Chukkali et al., 2023; Peixoto et al., 2023; Valusová and Schwarz, 2023), and this pattern also appears within musical populations (Luszuk et al., 2024; Philippe et al., 2019). For example, Philippe et al. (2019) showed that college music students performed worse on a number of well-being metrics than amateur musicians, while Luszuk et al. (2024) further demonstrated a direct association between music PA and reduced well-being. These findings suggest that greater performance-related stress is consistently associated with lower SWB among music performance students. Accordingly, we propose:

*H1*: PA is negatively associated with SWB among music performance students.

### Performance anxiety, psychological resilience, and subjective well-being

In demanding stage and evaluative contexts, music performance students often report elevated levels of PA, which is frequently regarded as a depleting stressor (Braden et al., 2015). According to resilience theory (Masten, 2001; Sarkar and Fletcher, 2014), individuals maintain functioning in stressful situations through emotion regulation and attentional control, and they recover quickly following setbacks. According to this viewpoint, high PA could be linked to greater difficulties in regulatory functioning, whereas higher PR is generally associated with more adaptive regulation and recovery. For instance, Osborne et al. (2014) found that as PA increased among music students, resilience and self-efficacy tended to be lower. In a similar vein, in a sample of nursing undergraduates, Berdida and Grande (2023) reported that situational anxiety was negatively related to resilience, and resilient students were more likely to exhibit emotional stability and constructive adaptability. These results imply that PA may be negatively correlated with PR in music performance circumstances.

Beyond its link with anxiety, PR is strongly related to positive psychological outcomes, particularly SWB. Fredrickson (2001) highlighted the close connection between resilience and positive emotional experiences during stressful situations, providing a basis for understanding the affective aspect of this association. Studies indicate that students with higher resilience typically report more positive emotional experiences and greater life satisfaction, whereas those with lower resilience are more prone to deficits in well-being (Wu et al., 2023; Xiang, 2026). For example, Xiang (2026) demonstrated a strong correlation between resilience and SWB in a college student sample. Extending this line of evidence, scholars have directly examined the interplay between anxiety/depression, resilience, and well-being. Chuning et al. (2024) reported that resilience was associated with a weaker negative relationship between anxiety/depression and well-being in addition to having a favorable correlation with well-being. Hartson et al. (2023) further reported significant associations of both stress and resilience with well-being among undergraduates. Although these studies were conducted in contexts beyond music performance, they collectively suggest a pattern relevant to the proposed indirect association. PR is consistently associated with higher well-being in the presence of stress or anxiety. In light of this logic, we propose the following hypothesis:

*H2*: PA is indirectly associated with SWB through PR.

### Moderating role of perfectionism

Multidimensional perfectionism theory distinguishes achievement striving from critical evaluations of performance, providing a framework for understanding individual differences in psychological adjustment (Stoeber and Otto, 2006). In music contexts, striving for perfection has been associated with greater effort and achievement (Stoeber and Eismann, 2007), while students with lower resilience have reported greater negative reactions to imperfection (Arbinaga, 2023). These findings suggest that personal standards and responses to perceived shortcomings are relevant to psychological adjustment among music students facing performance demands.

Resilience theory emphasizes the role of psychological resources in adaptation under stress (Masten, 2001; Sarkar and Fletcher, 2014). Together with multidimensional perfectionism theory, this perspective suggests that the association between PA and PR may differ according to personal standards and self-critical evaluations. The achievement orientation associated with AP may correspond to a weaker negative PA-PR association, whereas the pressure associated with demanding standards may correspond to a stronger negative association. For MP, perceived shortcomings may make anxiety more closely associated with negative evaluations of coping capacity. These considerations motivate examining AP and MP as potential moderators of the PA-PR association.

For the PR-SWB association, personal standards and perceived discrepancies provide a context for evaluating emotional experiences and life satisfaction. At higher AP, PR may be more strongly associated with SWB because coping capacity is particularly relevant to demanding goals; alternatively, stringent criteria for achievement may constrain this association. At higher MP, PR may likewise be more strongly associated with SWB in the context of perceived shortcomings, while persistent dissatisfaction provides a competing basis for a weaker association. These possibilities suggest that the PR-SWB association may vary across levels of both perfectionism dimensions.

The 2 × 2 model of perfectionism emphasizes that different combinations of the two dimensions may correspond to different patterns of psychological adjustment (Gaudreau and Thompson, 2010). This perspective suggests that the implications of high standards depend partly on accompanying self-critical evaluations. Extending this framework to the proposed model, the moderating role of AP may depend on MP, and vice versa. The PA-PR association concerns anxiety and perceived coping capacity, whereas the PR-SWB association concerns coping capacity and subjective well-being, allowing for potentially different patterns of joint moderation. We therefore propose the following hypotheses:

*H3*: AP and MP moderate the association between PA and PR, and their joint effect further conditions this pathway.

*H4*: AP and MP moderate the association between PR and SWB, and their joint effect further conditions this pathway.

In Figure 1, the suggested research model is displayed.

**Figure 1:**
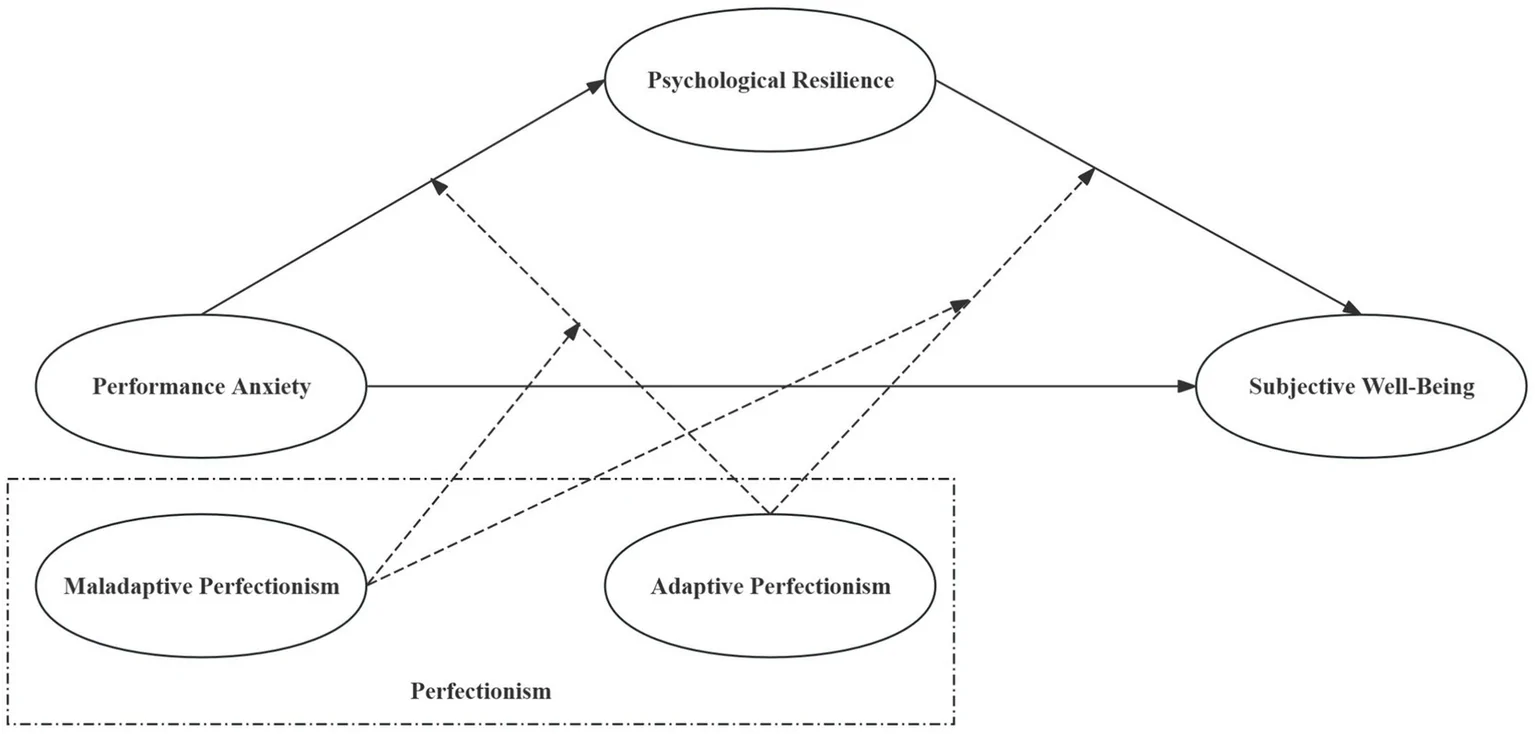
Research model.

## Methods

### Sample and data collection

The study was approved by the Ethics Committee of Zhangjiajie College (Approval No. 26052068), and all procedures complied with relevant ethical standards for human subjects research. Participation was voluntary, and informed consent was obtained before questionnaire completion. For participants under 18 years old, parental or legal guardian consent and participant assent were obtained prior to participation. Using convenience sampling, university music performance students from six universities in Hunan Province, China (including three public undergraduate universities and three private undergraduate universities) were recruited through Questionnaire Star^1^ between April and June 2026. The distribution of participants across institutions is presented in Supplementary Table S1. Participants varied in grade level, age, and daily practice duration. The questionnaire contained 72 questions, comprising 67 scale items and five demographic questions. The recruitment target was 864 participants (Kline, 2016). Of the 969 questionnaires collected, those with completion rates below 80% or more than 80% of responses concentrated at the same extreme option were excluded. The final sample comprised 946 valid responses, yielding a valid-response rate of 97.6%. Demographic characteristics are presented in Table 1. Independent-samples t-tests and one-way ANOVAs showed no significant differences in SWB across gender, grade level, age group, or daily practice duration. The primary models were estimated without demographic covariates.

**Table 1:** Demographic information of the participants.

Demographic characteristics	Category	Frequency	Percentage
Gender	Male	393	41.543%
	Female	553	58.457%
Grade level	Freshmen	240	25.37%
	Sophomores	246	26.00%
	Juniors	229	24.21%
	Seniors	231	24.42%
Age	Under 18 years old	241	25.48%
	18–22	415	43.87%
	Over 22 years old	290	30.65%
Hours of daily practice	Under 1 h	150	15.86%
	1–3 h	458	48.41%
	Over 3 h	338	35.73%

### Measurement instruments

PA was assessed using the complete 40-item Kenny Music Performance Anxiety Inventory (K-MPAI) (Kenny, 2023). The original instrument contains items covering multiple aspects of music PA; however, because the present study focused on overall PA rather than its specific dimensions, the scale was treated as an overall construct in the subsequent analyses. All 40 items were administered to participants, and responses were rated on a 7-point Likert scale coded from 1 to 7. Eight items were reverse-scored according to the original scoring procedure. Previous research has demonstrated satisfactory reliability and validity of the Chinese version (Cui et al., 2024). The initial 40-item single-factor model showed suboptimal overall fit (*χ*^2^/df = 5.485, RMSEA = 0.069, TLI = 0.875, CFI = 0.881). A standardized factor loading of 0.70 was adopted as the item-retention criterion in this study, drawing on methodological guidance describing this level as desirable (Cheung et al., 2024). Seven items (Items 2, 5, 8, 9, 23, 29, and 33) had loadings below this criterion (0.541–0.670) and were removed. These items address interpersonal trust, familial anxiety, and parental responsiveness or support; their exclusion narrowed the coverage of these background domains. The excluded items and their standardized factor loadings are presented in Supplementary Table S2. Accordingly, 33 items were retained for the final measurement model and subsequent analyses. The mean of the retained items was used to calculate the PA score, with higher scores indicating greater PA. The retained items demonstrated excellent internal consistency in the present study (Cronbach’s *α* = 0.976). The final measurement model also showed acceptable fit (*χ*^2^/df = 2.857, RMSEA = 0.044, TLI = 0.959, CFI = 0.963, GFI = 0.918, AGFI = 0.902, IFI = 0.963), supporting the fit of the retained 33-item model in this sample. Sensitivity analyses using the complete 40-item PA score were conducted to assess the robustness of the main findings to item exclusion.

The 10-item Connor-Davidson Resilience Scale (Campbell-Sills and Stein, 2007) was used to measure PR. Responses were recorded on a five-point scale coded from 1 to 5, with higher scores indicating greater resilience. The PR score was calculated as the mean of the 10 items. The Chinese version has previously been used among college students (Su and He, 2023). Cronbach’s alpha for this study was 0.907. The single-factor CFA model showed satisfactory fit (*χ*^2^/df = 1.885, RMSEA = 0.031, TLI = 0.991, CFI = 0.993, GFI = 0.986, AGFI = 0.978, IFI = 0.993).

We used the Well-Being Index Scale (WBIS) to measure SWB (Campbell, 1976). The Chinese version has shown satisfactory internal consistency in a student sample (Cheng et al., 2025). The nine items that make up the WBIS comprise eight General Affect Index items and one Life Satisfaction item. On a 7-point Likert scale, participants gave their answers. The SWB score was calculated by adding the mean of the eight General Affect Index items to the Life Satisfaction item score multiplied by 1.1. Higher total scores indicate more SWB; the range is 2.1 (least happy) to 14.7 (most happy). Cronbach’s alpha in this study was 0.918, and the single-factor CFA model showed satisfactory fit (*χ*^2^/df = 2.678, RMSEA = 0.042, TLI = 0.987, CFI = 0.990, GFI = 0.983, AGFI = 0.971, IFI = 0.990).

The Chinese eight-item Short Almost Perfect Scale (SAPS) (Rice et al., 2014; Song et al., 2023) was used to gauge perfectionism. Eight items make up the scale, which covers two dimensions: MP (Discrepancy subscale) and AP (Standards subscale). A 7-point Likert scale ranging from 1 (strongly disagree) to 7 (strongly agree) was used to grade the items, and higher scores denoted higher levels of the corresponding perfectionism dimension. Each subscale contains four items, and AP and MP scores were calculated separately as the means of their respective items. Cronbach’s alpha in this study was 0.852 for MP and 0.879 for AP. The CFA models showed satisfactory fit (AP: *χ*^2^/df = 2.415, RMSEA = 0.039, TLI = 0.996, CFI = 0.999, GFI = 0.997, AGFI = 0.987, IFI = 0.999; MP: *χ*^2^/df = 2.417, RMSEA = 0.039, TLI = 0.995, CFI = 0.999, GFI = 0.999, AGFI = 0.987, IFI = 0.999).

### Data analysis

Statistical analyses were conducted using IBM SPSS Statistics 26.0 and AMOS 29.0. The measurement model was evaluated through CFA and standard reliability and validity criteria, and potential common method bias (CMB) was assessed using Harman’s single-factor test and the common latent factor method. Descriptive statistics, Pearson correlations, and demographic differences in SWB were subsequently examined. All continuous focal variables were standardized prior to PROCESS analyses to facilitate interpretation of the interaction terms, and the reported regression coefficients were based on these standardized variables. PROCESS Model 4 with 5,000 bootstrap resamples was used to test the indirect association through PR, whereas Model 72 examined the separate and joint moderating roles of AP and MP, followed by conditional-effect analyses. Conditional indirect effects at the mean and ±1 SD of AP and MP and their pairwise contrasts were evaluated using 5,000 bootstrap resamples. These contrasts assessed differences across moderator combinations because the nonlinear indirect-effect function in Model 72 cannot be summarized by a single linear index of moderated mediation. Sensitivity analyses repeated Models 4 and 72 using the complete 40-item PA mean score instead of the 33-item score, with all other analytic settings unchanged.

## Results

### Reliability test

Before testing the focal models, the psychometric properties of the study measures were evaluated. Cronbach’s α values ranged from 0.852 to 0.976, and composite reliability values ranged from 0.900 to 0.977, with all coefficients exceeding the recommended threshold of 0.70. AVE values ranged from 0.544 to 0.734, all above 0.50, supporting satisfactory internal consistency and convergent validity (Brown, 2002; Henseler et al., 2015).

### Discriminant validity test

Discriminant validity for PA, PR, and SWB was evaluated using the HTMT criterion (Table 2). All values were below the recommended cutoff of 0.90, indicating adequate distinction among the three constructs (Henseler et al., 2015).

**Table 2:** Heterotrait-Monotrait criterion.

Variable	SWB	PA	PR
SWB			
PA	0.210		
PR	0.595	0.104	

PA, performance anxiety; SWB, subjective well-being; PR, psychological resilience.

### Confirmatory factor analysis

CFA was conducted to evaluate a five-factor measurement model comprising PA, PR, SWB, AP, and MP, with the two perfectionism dimensions represented as separate factors. The model showed satisfactory fit, with *χ*^2^/df = 1.896, RMSEA = 0.031, and all incremental and goodness-of-fit indices exceeding 0.90 (TLI = 0.964, CFI = 0.966, GFI = 0.911, AGFI = 0.901, IFI = 0.966) (Ji et al., 2024; Schmitt, 2011).

### Test for common method bias

CMB was assessed using Harman’s single-factor test and a common latent factor (CLF) model. Six factors had eigenvalues greater than 1, with the first factor accounting for 33.93% of the variance, below the 40% threshold (Podsakoff and Organ, 1986). The CLF model did not significantly improve fit over the baseline measurement model (*p* > 0.05). These assessments provided limited reassurance regarding common method variance, although method-related bias in the observed associations remains possible.

### Descriptive statistics and correlations

As shown in Table 3, absolute skewness and kurtosis values were below 2 and 7, respectively (Kline et al., 2011). Analysis of correlations revealed PA was significantly and negatively correlated with PR and SWB. AP and MP also displayed significant associations with several core variables.

**Table 3:** Descriptive statistics and correlations.

Variable	M ± SD	SK	Kur	PA	PR	SWB	AP	MP
PA	4.456 ± 1.255	−0.832	−0.404	1				
PR	3.775 ± 0.681	−1.044	1.945	−0.097**	1			
SWB	10.761 ± 2.432	−0.675	0.446	−0.196***	0.518***	1		
AP	4.971 ± 1.306	−0.889	0.478	0.399***	0.249***	0.171***	1	
MP	4.477 ± 1.387	−0.341	−0.523	0.490***	−0.121***	−0.306***	0.249***	1

M, mean; SD, standard deviation; SK, skewness; Kur, kurtosis; *** *p* < 0.001; ** *p* < 0.01; AP, adaptive perfectionism; MP, maladaptive perfectionism; PA, performance anxiety; SWB, subjective well-being; PR, psychological resilience.

### Mediation analysis

PROCESS Model 4 was used to examine the indirect association between PA and SWB through PR. As shown in Table 4 and Figure 2, PA was significantly negatively associated with PR. When PA and PR were simultaneously included in the model, PA remained significantly negatively associated with SWB, whereas PR was significantly positively associated with SWB. The model explained 29.0% of the variance in SWB.

**Table 4:** Mediation analysis of PR in the association between PA and SWB.

Predictor	Outcome	*R*	*R^2^*	*F*	*β*	*SE*	*t*	95% CI
PA	PR	0.097	0.009	8.998**	−0.097	0.032	−3.000**	[−0.161, −0.034]
PA	SWB	0.538	0.290	192.284***	−0.147	0.028	−5.342***	[−0.201, −0.093]
PR					0.504	0.028	18.260***	[0.449, 0.558]

*** *p* < 0.001; ** *p* < 0.01; PA, performance anxiety; SWB, subjective well-being; PR, psychological resilience.

**Figure 2:**
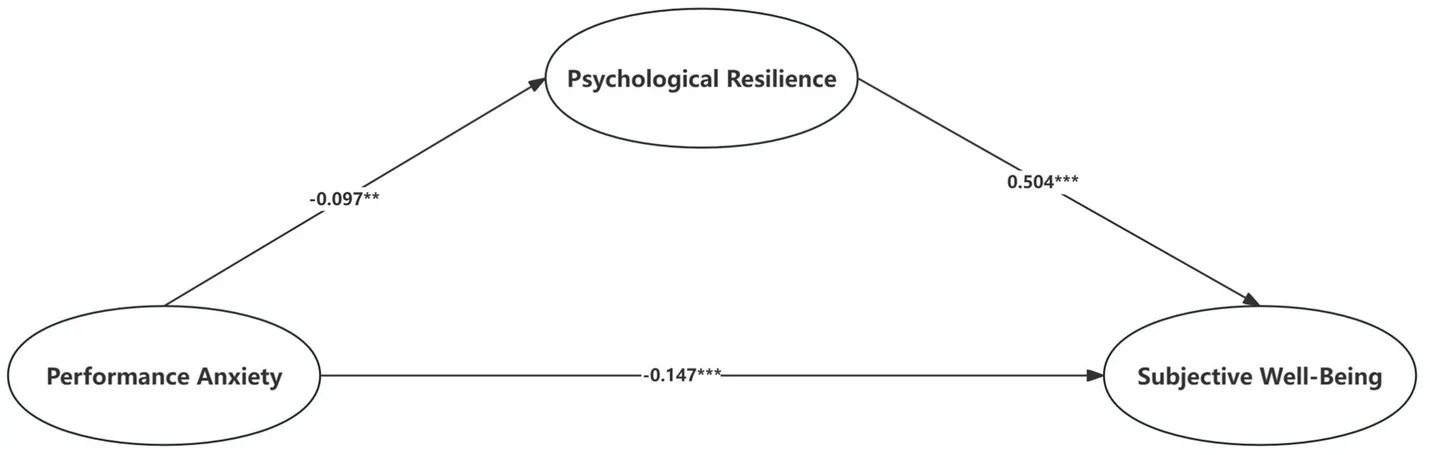
Results of the mediation model. *** *p* < 0.001; ** *p* < 0.01.

Bootstrap analysis with 5,000 resamples showed that the indirect association through PR was significant. As shown in Table 5, the indirect and direct associations were both significant.

**Table 5:** Estimates of total, direct, and indirect associations between PA and SWB through PR.

Effect Type	*β*	SE/Boot SE	95% CI
Total effect	−0.196	0.032	[−0.259, −0.134]
Direct effect	−0.147	0.028	[−0.201, −0.093]
Indirect effect	−0.049	0.023	[−0.097, −0.006]

### Moderation analysis

PROCESS Model 72 was used to examine the moderating roles of AP and MP on the two component paths. For the PA-PR association, the PA × AP and PA × MP interactions were significant, whereas the PA × AP × MP interaction was not significant (Table 6). Thus, the separate moderating roles of AP and MP were supported, but their joint moderating role on this path was not, providing partial support for H3. Figures 3A,C,E illustrate the conditional PA-PR associations across AP levels at low, mean, and high MP, respectively.

**Table 6:** Moderation analysis of adaptive perfectionism and maladaptive perfectionism.

Predictor	Outcome	*R*	*R^2^*	*F*	*B*	*SE*	*t*	95% CI
PA	PR	0.433	0.188	30.950***	−0.125	0.037	−3.373**	[−0.198, −0.052]
AP					0.432	0.041	10.583***	[0.352, 0.512]
PA × AP					0.228	0.031	7.452***	[0.168, 0.288]
MP					−0.109	0.037	−2.974**	[−0.181, −0.037]
PA × MP					0.131	0.027	4.815***	[0.077, 0.184]
AP × MP					−0.098	0.037	−2.673**	[−0.169, −0.026]
PA × AP × MP					0.030	0.021	1.426	[−0.011, 0.070]
PA	SWB	0.634	0.402	78.844***	−0.093	0.032	−2.867**	[−0.157, −0.029]
PR					0.507	0.031	16.462***	[0.447, 0.568]
AP					0.156	0.032	4.914***	[0.094, 0.218]
PR × AP					0.075	0.021	3.603***	[0.034, 0.116]
MP					−0.226	0.031	−7.371***	[−0.286, −0.166]
PR × MP					0.070	0.025	2.792**	[0.021, 0.119]
AP × MP					0.081	0.021	3.906***	[0.040, 0.122]
PR × AP × MP					−0.039	0.014	−2.822**	[−0.067, −0.012]

*** *p* < 0.001; ** *p* < 0.01; AP, adaptive perfectionism; MP, maladaptive perfectionism; PA, performance anxiety; SWB, subjective well-being; PR, psychological resilience.

**Figure 3:**
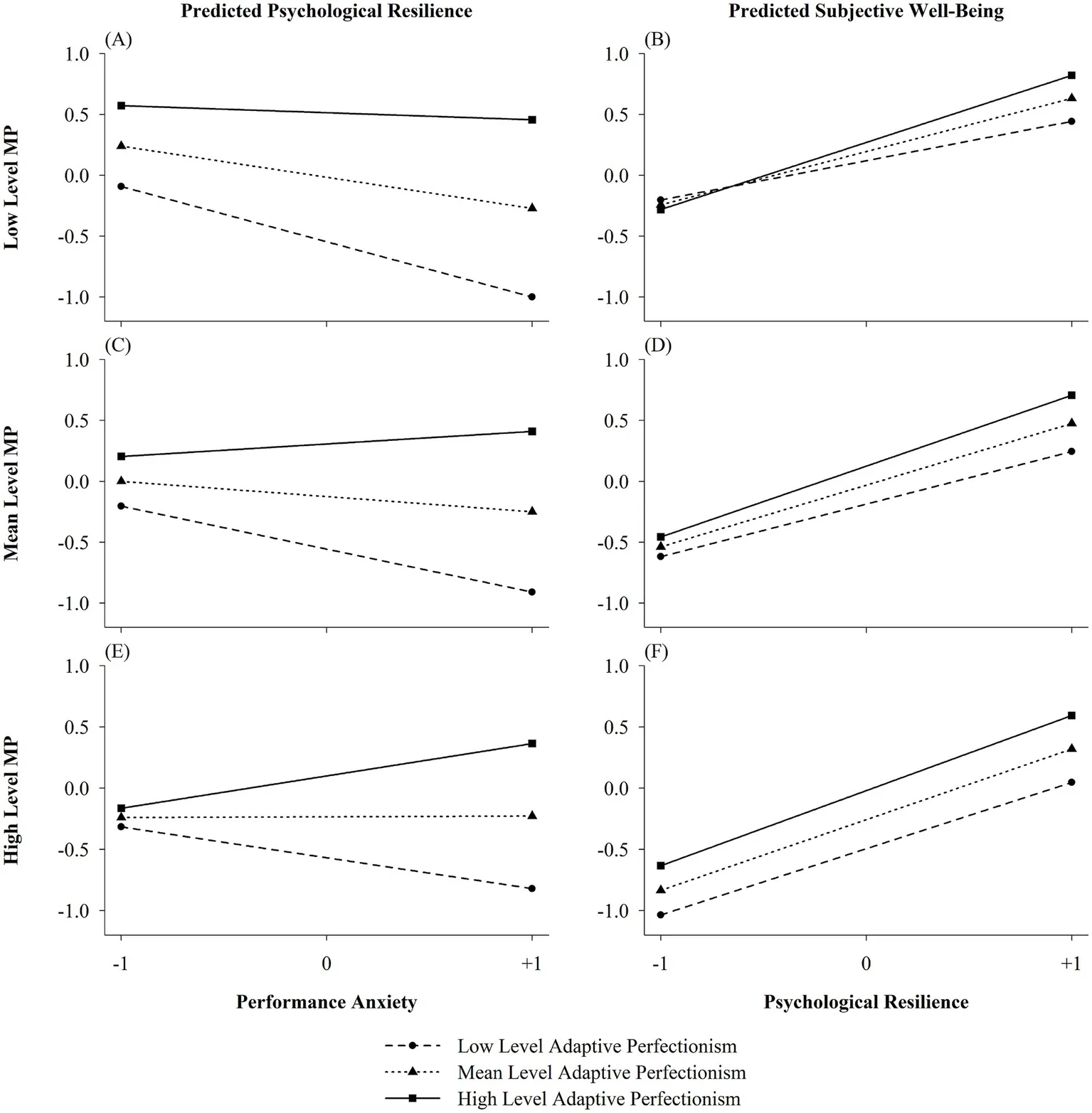
Conditional associations across levels of adaptive and maladaptive perfectionism. Panels **(A)**, **(C)**, and **(E)** show the associations between performance anxiety and psychological resilience; panels **(B)**, **(D)**, and **(F)** show the associations between psychological resilience and subjective well-being. Rows represent low, mean, and high maladaptive perfectionism (MP), respectively. Lines represent low, mean, and high adaptive perfectionism. Low and high levels correspond to one standard deviation below and above the mean. All plotted values are based on standardized variables, and lines represent model-predicted values. Performance anxiety is held at its mean in Panels **(B)**, **(D)**, and **(F)**.

For the PR-SWB association, both two-way interactions and the PR × AP × MP interaction were significant, supporting H4. Conditional-effect analyses indicated that PR remained positively associated with SWB across all examined AP and MP combinations. The negative three-way interaction indicated that the additional strengthening associated with either perfectionism dimension diminished as the other increased, without reversing the positive PR-SWB association. These conditional associations are illustrated in Figures 3B,D,F for low, mean, and high MP, respectively.

### Conditional indirect effects

The conditional indirect association between PA and SWB through PR was examined across combinations of AP and MP (Table 7). When AP was low, the indirect association was significantly negative at all examined MP levels. At mean AP, it was significantly negative at low and mean MP but non-significant at high MP. When AP was high, it was non-significant at low and mean MP and significantly positive at high MP.

**Table 7:** Conditional indirect effects of PA on SWB through PR at different levels of AP and MP.

AP level	MP level	Indirect effect	Boot SE	95% Bootstrap CI
Low	Low	−0.147	0.031	[−0.214, −0.093]
	Mean	−0.153	0.033	[−0.217, −0.088]
	High	−0.136	0.051	[−0.234, −0.032]
Mean	Low	−0.112	0.020	[−0.151, −0.074]
	Mean	−0.063	0.027	[−0.114, −0.007]
	High	0.003	0.047	[−0.082, 0.103]
High	Low	−0.031	0.026	[−0.080, 0.024]
	Mean	0.060	0.042	[−0.014, 0.149]
	High	0.161	0.069	[0.038, 0.303]

Low, mean, and high refer to −1 SD, the mean, and +1 SD, respectively. AP, adaptive perfectionism; MP, maladaptive perfectionism; PA, performance anxiety; PR, psychological resilience; SWB, subjective well-being.

Bootstrap contrasts further examined these differences while holding one moderator constant and when both moderators varied. At mean MP, the indirect effect was greater at high than at low AP [difference = 0.213, 95% bootstrap CI (0.118, 0.317)]. At mean AP, it was greater at high than at low MP [difference = 0.115, 95% bootstrap CI (0.035, 0.209)]. Comparing the two joint configurations, the indirect effect was greater at high AP and high MP than at low AP and low MP [difference = 0.308, 95% bootstrap CI (0.172, 0.469)].

### Sensitivity analyses

Models 4 and 72 were re-estimated using the complete 40-item PA score. In Model 4, the negative direct and indirect associations between PA and SWB remained significant. In Model 72, the focal regression paths, interaction terms, and all nine conditional indirect effects retained their directions and statistical significance patterns, with similar effect estimates. All pairwise contrasts that were significant in the main analysis remained significant in the sensitivity analysis. Two additional contrasts became significant, with the lower bounds of their 95% bootstrap confidence intervals slightly above zero. These findings support the robustness of the main conclusions to item exclusion. Detailed results are presented in Supplementary Tables S3–S7.

## Discussion

The significant negative association between PA and SWB among music performance students is consistent with the findings of Luszuk et al. (2024). In music performance contexts, students may worry that mistakes are unavoidable even with careful preparation, fear negative audience reactions, or believe that one unsatisfactory performance could jeopardize future opportunities. Repeated self-doubt and fear of failure are often associated with lower self-worth, greater frustration, and less positive perceptions of learning and life (Raedeke et al., 2021). Significant emotional and physiological reactions, such as elevated heart rate, tense muscles, insomnia, or ongoing thoughts about performance adequacy, are also commonly linked to PA. These psychological and physiological reactions are also commonly associated with lower well-being, enjoyment, and satisfaction during music study and performance (Muñoz and Rentería, 2023). These findings identify performance-related anxiety as a relevant focus for psychological assessment and support in music education.

A significant indirect association between PA and SWB was observed through PR. According to Hartson et al. (2023), in a group of college students, resilience was favorably correlated with well-being while anxiety was adversely correlated with it. The trend found in the current investigation is indirectly supported by these results. In this study, from the perspective of resilience theory (Masten, 2001; Sarkar and Fletcher, 2014), PA may be accompanied by lack of motivation during preparation and performance, pessimistic expectations about the future, or doubts about the value of long-term commitment. Such experiences may be associated with fewer positive emotions and lower overall satisfaction. Students with higher resilience may be more likely to identify stress cues and reappraise them, stabilize attention and emotion, persist in pursuing musical goals, and recover more quickly from fluctuations in practice and performance, while maintaining hope and a sense of value (Yubero et al., 2023). Moreover, PA may also be accompanied by social insecurity, such as difficulty trusting others during collaboration, discomfort with teacher feedback, or avoidance of performance opportunities due to fear of evaluation. Such experiences are closely linked to reduced feelings of support and satisfaction. By contrast, students with higher resilience may be more likely to maintain positive interpersonal relationships, draw on available support, and appraise challenges more adaptively. These characteristics may correspond to greater positive emotions and satisfaction in performance contexts (Li et al., 2024). Overall, the findings identify PR as a relevant psychological resource associated with well-being under performance-related pressure. However, the cross-sectional design does not establish the temporal or causal ordering of these associations.

AP and MP each moderated the association between PA and PR, whereas their joint moderating effect was not significant. In music performance training, high personal standards may accompany both concern about meeting demanding expectations and an active orientation toward preparation and problem solving. Personal standards can coexist with elevated music PA and active coping (McNeil et al., 2022). Students with higher AP may therefore experience anxiety alongside a readiness to address performance demands, offering a possible explanation for the more positive conditional PA-PR association. The positive AP-PA correlation contrasts with the negative association reported among preservice music teachers (He and Ding, 2025). The importance attached to technical accuracy and successful performance may make falling short of personal expectations particularly concerning, even among students who report stronger coping capacity. For MP, the more positive conditional PA-PR association qualifies the expectation that perceived performance discrepancies would intensify the negative association between anxiety and resilience. Self-critical perfectionism is associated with daily stress, avoidant coping, and lower perceived social support (Dunkley et al., 2003), suggesting several contextual factors that may be relevant to this unexpected pattern. In demanding rehearsals and assessments, the relationship between anxiety and perceived coping capacity may depend partly on how students respond to perceived shortcomings and the support available to them. Examining these factors could clarify the circumstances under which higher MP accompanies a weaker negative or positive PA-PR association. The non-significant three-way interaction places a boundary on the proposed joint moderation: in this sample, the individual moderating roles of AP and MP were more clearly supported than their combined role. Future research across routine practice and high-stakes assessments could examine whether their joint moderation varies with the intensity of performance demands.

Both AP and MP moderated the positive association between PR and SWB, and their joint moderating effect was also significant. In music performance training, students with high personal standards may attach particular importance to progress toward demanding musical goals. Perfectionistic striving is associated with greater effort and achievement among young musicians (Stoeber and Eismann, 2007). For students with higher AP, perceived capacity to handle mistakes, criticism, and difficult assessments may therefore be particularly relevant to whether these goals feel manageable and satisfying. At higher MP, dissatisfaction with perceived performance discrepancies may make resilience particularly relevant to well-being amid self-critical concerns. The association of perfectionistic concerns with psychological distress suggests that perceived coping capacity may be especially important to emotional experience and life satisfaction in this context (Stoeber and Otto, 2006). When high standards and substantial dissatisfaction coexist, however, students may place considerable weight on meeting demanding expectations while remaining focused on perceived shortcomings. Feeling capable of handling a difficult rehearsal or assessment may then coexist with dissatisfaction about technical errors or an imperfect performance. Such an emphasis on unmet expectations is consistent with the view that perfectionistic self-evaluation can depend heavily on attaining demanding standards (Shafran et al., 2002). At higher MP, difficulty regarding achievement as satisfactory may therefore limit the additional strengthening of the PR-SWB association associated with AP. This interpretation is consistent with the 2 × 2 model’s emphasis on psychological adjustment across combinations of personal standards and evaluative concerns (Gaudreau and Thompson, 2010). The PR–SWB association remained positive across all examined combinations. Personal standards and perceived discrepancies may jointly shape whether students regard their musical progress as satisfactory, making their combination particularly relevant to how coping capacity relates to well-being. The PA–PR association concerns anxiety and perceived capacity to manage difficulties, whereas the PR–SWB association additionally involves satisfaction with those experiences. This distinction offers a possible explanation for the joint moderation observed on the latter path. Future research could assess satisfaction with musical progress alongside coping appraisals to examine this proposed distinction.

The conditional indirect effects further suggest that anxiety in music performance should be understood in relation to students’ goal pursuit and self-evaluation. In contexts involving repeated practice and public evaluation, anxiety may accompany doubts about coping capacity or the importance attached to performance goals. The positive associations of personal standards with both performance anxiety and active coping provide support for the latter possibility (McNeil et al., 2022). When high standards coexist with substantial perceived performance discrepancies, students may attend to both the importance of a performance and the gap between their actual and expected performance. The importance attached to goals may be associated with preparation and effort, while attention to discrepancies may accompany worry, allowing anxiety and coping confidence to coexist. This offers one possible explanation for the positive indirect association observed at high AP and high MP: students reporting greater anxiety may also perceive themselves as capable of managing training demands, and this perceived capacity is associated with greater well-being. In combinations where the negative indirect association was significant, anxiety may instead be more closely connected to negative appraisals of the manageability of difficulties and personal coping capacity. Variation in the direction of the indirect association may therefore involve how students appraise performance demands and their coping resources. Meanwhile, the negative direct association between PA and SWB remained significant, suggesting that coping confidence may coexist with the emotional burden associated with anxiety. Future research could examine perceived performance importance, coping confidence, and actual preparation behaviors to assess whether these factors help explain the indirect associations across different perfectionism combinations.

## Implications

### Theoretical implications

First, drawing on resilience theory and multidimensional perfectionism theory, this study developed an integrative framework linking PA, PR, perfectionism, and SWB in high-evaluation music contexts. The conditional model explained 40.2% of the variance in SWB. Second, the significant indirect association through PR extends the application of resilience theory by highlighting the statistical linkage between performance-related anxiety, psychological resources, and well-being. Finally, by simultaneously examining AP and MP, the study moves beyond a single-dimensional view of perfectionism. The findings indicate separate moderating patterns for both perfectionism dimensions on the PA-resilience association, while a significant higher-order conditional pattern emerged only for the resilience-well-being association. This distinction provides a more nuanced extension of the configurational perspective of multidimensional perfectionism.

### Practical implications

For music performance students, the findings highlight the value of considering performance anxiety alongside perceived coping capacity and self-evaluation. Students with high personal standards may find it useful to reflect on both their musical ambitions and the anxiety accompanying these expectations. During rehearsals, examinations, and performances, relevant questions include how manageable performance demands feel and how strongly satisfaction depends on meeting personal standards. Students who combine high standards with substantial dissatisfaction may also consider whether they can acknowledge progress alongside remaining technical difficulties. These reflections may help students articulate their concerns when seeking academic or psychological support.

For higher education institutions, psychological support for music performance students may be strengthened by considering PA, resilience, and perfectionistic tendencies together. Advising and counselling conversations could include students’ perceived ability to manage mistakes and criticism, their satisfaction with musical progress, and their interpretations of unmet expectations. Strong achievement striving should be accompanied by attention to possible anxiety, while students reporting both high standards and pronounced dissatisfaction warrant consideration of coping confidence and well-being together. Support needs should be assessed individually across perfectionism levels, including combinations for which the conditional indirect association was nonsignificant. These findings can inform the focus of assessment and consultation; the effectiveness of any resulting support strategies requires separate evaluation.

## Limitations and future research

First, self-report measures were the only ones used in this investigation. Although previous research has shown these instruments to be highly valid and reliable, subjective views and social desirability effects may still have an impact on the data, which could reduce the accuracy of conclusions on variable relationships. The common method bias assessments provided some reassurance, although Harman’s single-factor test has limited sensitivity and the common latent factor approach is dependent on model specification. Some method-related influence on the observed associations may therefore remain. Future research could incorporate interviews or other-report measures and separate the measurement of predictors and outcomes in time to reduce subjective bias, enhance data credibility, and strengthen model robustness.

Second, the study sample consisted of university music performance students, who face unique high-pressure conditions in training and stage performance. As such, the findings primarily apply to this population. Future studies could extend the model to other high-stress groups, such as performers in other art forms or competitive athletes, to examine the cross-contextual stability of the relationships among PR, perfectionism, and well-being.

Third, the cross-sectional methodology did not permit causal inference and simply identified relationships between PA, PR, perfectionism, and well-being. To dynamically monitor changes in variables, future studies may use experimental or longitudinal approaches. For example, diary-based or multi-wave studies during examination periods and regular weeks could assess short-term and delayed associations, further testing the temporal stability of perfectionism’s moderating role and deepening the understanding of psychological adaptation mechanisms.

In addition, participants were recruited using convenience sampling from multiple universities in China. The non-probability sampling technique may restrict the sample’s representativeness and the findings’ generalizability, despite the fact that the sample comprised students with a variety of practice and demographic characteristics. Future research could use stratified or multistage probability sampling across institutions and regions to improve population coverage.

## Conclusion

This study investigated the relationships among PA, PR, perfectionism, and SWB in a sample of university music performance students. In summary, PA was negatively associated with SWB, and a significant indirect association was observed through PR. AP and MP separately moderated both focal associations, whereas their joint conditional patterns differed across the two pathways. No significant three-way interaction was observed for the PA-resilience association, while a significant negative three-way interaction emerged for the resilience-well-being association. These findings highlight the distinct and configurational roles of perfectionism in the psychological adjustment of music performance students. The results should be viewed as statistical connections rather than proof of causal or temporal processes because of the cross-sectional approach.

## Data Availability

The raw data supporting the conclusions of this article will be made available by the authors, without undue reservation.

## Footnotes

1
www.sojump.com
