# Supplementary material for: Exploring the relationship between performance anxiety and subjective well-being in music performance students: the role of psychological resilience and perfectionism
Source: Front Psychol. 2026 Oct 26;17:1967733. doi: 10.3389/fpsyg.2026.1967733 (PMC13745355; doi:10.3389/fpsyg.2026.1967733)
Supplement: Supplementary file 1 [file Supplementary_file_1.doc]

# Appendix

Appendix Table S 1 Distribution of Participants Across Institutions

| Institution Code | Institution Type | Number of Participants |
| --- | --- | --- |
| University 1 | Public undergraduate university | 129 |
| University 2 | Public undergraduate university | 189 |
| University 3 | Public undergraduate university | 218 |
| University 4 | Private undergraduate university | 111 |
| University 5 | Private undergraduate university | 153 |
| University 6 | Private undergraduate university | 146 |

Appendix Table S 2 Excluded K-MPAI Items, Standardized Factor Loadings

| number | Item content | Standardized factor loading |
| --- | --- | --- |
| 2 | I find it easy to trust others. | 0.641 |
| 5 | Excessive worrying is a characteristic of my family. | 0.670 |
| 8 | I find it difficult to depend on others. | 0.618 |
| 9 | My parents were responsive to my needs most of the time. | 0.541 |
| 23 | My parents were always good listeners. | 0.613 |
| 29 | One or both of my parents were sometimes overly anxious. | 0.630 |
| 33 | My parents encouraged me to try new things. | 0.634 |

Appendix Table S 3 Regression Results for the Simple Mediation Model Using the Complete 40-Item Performance Anxiety Score

| Outcome | Predictor | B | SE | *t* | *p* | 95% CI |
| --- | --- | --- | --- | --- | --- | --- |
| Psychological Resilience | Performance Anxiety | -0.094 | 0.032 | -2.892 | 0.004 | [-0.157, -0.030] |
| Subjective Well-Being | Performance Anxiety | -0.146 | 0.028 | -5.307 | <0.001 | [-0.200, -0.092] |
|  | Psychological Resilience | 0.504 | 0.028 | 18.285 | <0.001 | [0.450, 0.558] |

Appendix Table S 4 Total, Direct, and Indirect Effects Using the Complete 40-Item Performance Anxiety Score

| Effect | Estimate | SE / Bootstrap SE | 95% CI |
| --- | --- | --- | --- |
| Total Effect of Performance Anxiety on Subjective Well-Being | -0.194 | 0.032 | [-0.256, -0.131] |
| Direct Effect of Performance Anxiety on Subjective Well-Being | -0.146 | 0.028 | [-0.200, -0.092] |
| Indirect Effect Through Psychological Resilience | -0.047 | 0.023 | [-0.092, -0.005] |

Appendix Table S 5 Regression Results for the Moderated Mediation Model Using the Complete 40-Item Performance Anxiety Score

| Outcome | Predictor | B | SE | *t* | *p* | 95% CI |
| --- | --- | --- | --- | --- | --- | --- |
| Psychological Resilience | Performance Anxiety | -0.127 | 0.036 | -3.488 | 0.001 | [-0.199, -0.056] |
|  | Adaptive Perfectionism | 0.436 | 0.041 | 10.666 | <0.001 | [0.355, 0.516] |
|  | Performance Anxiety × Adaptive Perfectionism | 0.233 | 0.031 | 7.611 | <0.001 | [0.173, 0.293] |
|  | Maladaptive Perfectionism | -0.110 | 0.036 | -3.028 | 0.003 | [-0.182, -0.039] |
|  | Performance Anxiety × Maladaptive Perfectionism | 0.131 | 0.027 | 4.802 | <0.001 | [0.077, 0.184] |
|  | Adaptive Perfectionism × Maladaptive Perfectionism | -0.098 | 0.037 | -2.664 | 0.008 | [-0.169, -0.026] |
|  | Performance Anxiety × Adaptive Perfectionism × Maladaptive Perfectionism | 0.032 | 0.021 | 1.545 | 0.123 | [-0.009, 0.073] |
| Subjective Well-Being | Performance Anxiety | -0.094 | 0.032 | -2.931 | 0.003 | [-0.158, -0.031] |
|  | Psychological Resilience | 0.507 | 0.031 | 16.476 | <0.001 | [0.447, 0.568] |
|  | Adaptive Perfectionism | 0.157 | 0.032 | 4.939 | <0.001 | [0.094, 0.219] |
|  | Psychological Resilience × Adaptive Perfectionism | 0.076 | 0.021 | 3.620 | <0.001 | [0.035, 0.116] |
|  | Maladaptive Perfectionism | -0.226 | 0.031 | -7.422 | <0.001 | [-0.286, -0.167] |
|  | Psychological Resilience × Maladaptive Perfectionism | 0.070 | 0.025 | 2.804 | 0.005 | [0.021, 0.119] |
|  | Adaptive Perfectionism × Maladaptive Perfectionism | 0.081 | 0.021 | 3.879 | <0.001 | [0.040, 0.121] |
|  | Psychological Resilience × Adaptive Perfectionism × Maladaptive Perfectionism | -0.039 | 0.014 | -2.799 | 0.005 | [-0.067, -0.012] |

Appendix Table S 6 Conditional Indirect Effects Through Psychological Resilience Using the 33-Item and 40-Item Performance Anxiety Scores

| Adaptive Perfectionism Level | Maladaptive Perfectionism Level | 33-Item | | 40-Item | | |
| --- | --- | --- | --- | --- | --- | --- |
|  |  | Estimate | 95% Bootstrap CI | Estimate | Bootstrap SE | 95% Bootstrap CI |
| Low | Low | -0.147 | [-0.214, -0.093] | -0.148 | 0.031 | [-0.215, -0.094] |
|  | Mean | -0.153 | [-0.217, -0.088] | -0.156 | 0.033 | [-0.221, -0.093] |
|  | High | -0.136 | [-0.234, -0.032] | -0.142 | 0.050 | [-0.237, -0.038] |
| Mean | Low | -0.112 | [-0.151, -0.074] | -0.113 | 0.020 | [-0.154, -0.076] |
|  | Mean | -0.063 | [-0.114, -0.007] | -0.065 | 0.026 | [-0.114, -0.011] |
|  | High | 0.003 | [-0.082, 0.103] | 0.002 | 0.046 | [-0.084, 0.098] |
| High | Low | -0.031 | [-0.080, 0.024] | -0.031 | 0.026 | [-0.080, 0.025] |
|  | Mean | 0.060 | [-0.014, 0.149] | 0.062 | 0.042 | [-0.013, 0.150] |
|  | High | 0.161 | [0.038, 0.303] | 0.165 | 0.068 | [0.044, 0.308] |

Appendix Table S 7 Selected Pairwise Contrasts Between Conditional Indirect Effects Using the 33-Item and 40-Item Performance Anxiety Scores

| Contrast | 33-Item | | 40-Item | | |
| --- | --- | --- | --- | --- | --- |
|  | Difference | 95% Bootstrap CI | Difference | Bootstrap SE | 95% Bootstrap CI |
| High versus Low Adaptive Perfectionism at Mean Maladaptive Perfectionism | 0.213 | [0.118, 0.317] | 0.217 | 0.051 | [0.122, 0.322] |
| High versus Low Maladaptive Perfectionism at Mean Adaptive Perfectionism | 0.115 | [0.035, 0.209] | 0.115 | 0.044 | [0.034, 0.208] |
| High Adaptive-High Maladaptive Perfectionism versus Low Adaptive-Low Maladaptive Perfectionism | 0.308 | [0.172, 0.469] | 0.313 | 0.077 | [0.176, 0.477] |
| Mean Adaptive-Mean Maladaptive Perfectionism versus Low Adaptive-High Maladaptive Perfectionism | 0.073 | [-0.001, 0.144] | 0.077 | 0.037 | [0.002, 0.146] |
| High Adaptive-Mean Maladaptive Perfectionism versus Mean Adaptive-High Maladaptive Perfectionism | 0.057 | [-0.002, 0.113] | 0.060 | 0.029 | [0.002, 0.113] |
